# Optimizing plant density and nitrogen application to manipulate tiller growth and increase grain yield and nitrogen-use efficiency in winter wheat

**DOI:** 10.7717/peerj.6484

**Published:** 2019-02-26

**Authors:** Dongqing Yang, Tie Cai, Yongli Luo, Zhenlin Wang

**Affiliations:** 1 College of Agronomy, Shandong Agricultural University, Taian, Shandong, People’s Republic of China; 2 College of Agronomy, Northwest A&F University, Yangling, Shanxi, People’s Republic of China

**Keywords:** Nitrogen, Plant density, Nitrogen-use efficiency, Grain yield, Superior tiller group

## Abstract

The growth of wheat tillers and plant nitrogen-use efficiency (NUE) will gradually deteriorate in response to high plant density and over-application of N. Therefore, in this study, a 2-year field study was conducted with three levels of plant densities (75 ×10^4^plants ha^−1^, D1; 300 ×10^4^plants ha^−1^, D2; 525 ×10^4^plants ha^−1^, D3) and three levels of N application rates (120 kg N ha^−1^, N1; 240 kg N ha^−1^, N2; 360 kg N ha^−1^, N3) to determine how to optimize plant density and N application to regulate tiller growth and to assess the contribution of such measures to enhancing grain yield (GY) and NUE. The results indicated that an increase in plant density significantly increased the number of superior tillers and the number of spikes per m^2^(SN), resulting in a higher GY and higher partial factor productivity of applied N (PFP_N_). However, there was no significant difference in GY and PFP_N_ between plant densities D2 and D3. Increasing the N application rate significantly increased the vascular bundle number (NVB) and area (AVB), however, excess N application (N3) did not significantly improve these parameters. N application significantly increased GY, whereas there was a significant decrease in PFP_N_ in response to an increase in N application rate. The two years results suggested that increasing the plant density (from 75 ×10^4^plants ha^−1^to 336 ×10^4^plants ha^−1^) in conjunction with the application of 290 kg N ha^−1^N will maximize GY, and also increase PFP_N_(39.7 kg kg^−1^), compared with the application of 360 kg N ha^−1^N. Therefore, an appropriate combination of increased planting density with reduced N application could regulate tiller number and favor the superior tiller group, to produce wheat populations with enhanced yield and NUE.

## Introduction

Wheat is one of the most important food crops worldwide. In China, where wheat has accounted for more than 20% of the total sowing area in recent years, production has substantially increased during the past few decades (Chen et al., 2017). Nonetheless, owing to continual population growth, the demand for wheat will continue to increase. Moreover, arable areas have undergone a substantial decrease in extent due to urban expansion and environmental degradation (Wei et al., 2016), and wheat production currently faces severe constraints and difficult challenges (Li et al., 2016a). Therefore, it is essential to ensure food security through increasing yield per unit area, to enhance the total production on a diminishing area on cultivated farmland.

Tillering is an important agronomic trait in graminaceous crops such as wheat and rice, as it determines final spike number per m^2^ and plays an important role in determining grain yield (Naruoka et al., 2011; Wang et al., 2016b). When fewer tillers are produced, a smaller population is formed, resulting in yield reductions due to a smaller population of spikes per m^2^ (Mitchell et al., 2012; Moeller, Evers & Rebetzke, 2014). Conversely, an excess of surviving tillers can lead to a larger population, resulting in an increased risk of lodging risk due to reduced culm quality (Wang et al., 2009; Zheng et al., 2017). In addition, individual tillers exhibit heterogeneity. In this regard, Cai et al. (2014) reported decreases in grain number and kernel weight per spike as the tiller position shifts from low to high, and the flag leaves of low-position tillers have been shown to have a higher photosynthetic rate than high-position tillers (Xu et al., 2015a). There is also competition between low- and high-position tillers for limiting resources (Elhani et al., 2007; Ma et al., 2008). Early-emerging tillers generally intercept more radiation and shade the late-emerging tillers, and consequently dominate the asymmetric competition from the early growth stage (Moeller, Evers & Rebetzke, 2014). Late-emerging tillers generally make a minimal contribution to grain yield formation (Otteson et al., 2008), and indeed, these unproductive tillers can negatively affect grain yield by competing for resources such as nitrogen (N) and solar radiation (Richards et al., 2002; Berry et al., 2003). Hence, management of tiller emergence and heading, and utilization of early-emerging tillers, are key factors for the establishment of high-yield and high-efficiency populations.

The occurrence of tillers generally involves two developmental stages: the formation of axillary meristems in the leaf axil and the growth of tillers (Li et al., 2003; Bennett & Leyser, 2006). Tiller development is a result of complex interactions between endogenous signals and environmental factors. Internal factors regulating tiller emergence include numerous genetic factors (Xie, Mayes & Sparkes, 2016; Hyles et al., 2017), and endogenous hormones (Lin et al., 2016). External factors including light, plant density, and fertilizer application, have a significant effect on the growth of tillers (Assuero & Tognetti, 2010). The ratio of red to far-red light irradiance influences the outgrowth of tiller buds (Evers et al., 2006), whereas increasing planting density from 135 to 405 plants per m^2^ has been shown to significantly increase grain yield (Dai et al., 2014), although further increases in density do not affect grain yield. Although seeding rate has been shown to have no influence on main leaf development, it does have a considerable effect on tiller development (Chen et al., 2008). Increasing the seeding rate has been found to increase the proportion of yield obtained from the main spike and decreases the proportion from high-position tiller spikes (Otteson et al., 2008). Although low plant density produces a higher grain number and grain weight per spike (Li et al., 2016b), this is generally not sufficient to compensate for the lower spike density per m^2^ generated by a lower tiller density (Clerget et al., 2016). Therefore, an appropriate increase in plant density to balance yield component factors would appear to be an apposite agronomic management strategy for enhancing grain yield.

Nitrogen is an important and essential nutrient for all plants. N supply has considerable effects on plant growth in terms of the amount of biomass produced, the size and proportion of organs and their structure, and the progress of plant development (Lawlor, Lemaire & Gastal, 2001). N regulates rice tiller bud growth through regulating endogenous hormones and N metabolism (Liu et al., 2011). Nitrogen deficiency is associated with a reduced rate of leaf appearance and reduces the maximum rate of emergence for each tiller (Prystupa, Slafer & Savin, 2003), whereas an increase in N application rate increases tiller density and reduces tiller mortality (Ahmed et al., 2016). Increasing N application increases yield through an increase in grain yield in early-emerging tillers (Wang et al., 2016a). Furthermore, grain number per unit area is significantly increased in response to N application, mainly due to an increase in the contribution of grains produced by tillers (Terrile, Miralles & González, 2017). In China, farmers excessively apply N fertilizers because of their hope to sustain further grain yield increases, but grain yield does not keep synchronous increase with excessive N application (Meng et al., 2016). N application during the wheat growing season generally exceeds 320∼350 kg N ha^−1^; however, some farmers uses rates as high as 750 kg N ha^−1^ (Cui, Chen & Zhang, 2010; Lu et al., 2015; Zhang et al., 2015). Over-application of N contributes little to enhancing grain yield but can reduce nitrogen-use efficiency (NUE) and enhances the risk of environmental pollution (Cui, Chen & Zhang, 2010; Zhang et al., 2015; Mon et al., 2016; Tian et al., 2017). Therefore, achieving both high yield and high NUE simultaneously is a major challenge. Lu et al. (2014) have reported that optimal N management can optimize population quantity and quality to achieve high grain yield, and therefore a high-yielding wheat population can be obtained by amending N supply to regulate tiller development. Accordingly, N application rates should be calculated to produce the required quantity of tillers and modified over time to generate wheat crops with the desired NUE.

Although previous studies have either reported the effects of plant density on tiller development (e.g., tiller emergence, tiller mortality, and tiller production) or focused on N demand and supply, there have been few studies that have systematically investigated the relationship between these aspects, for example, the mechanisms underlying the differences in grain yield among different tiller spikes and how to manipulate tillers to establish high-yield and high-efficiency groups. Moreover, little is known regarding how plant density and N application interactively regulate tiller growth and wheat population development to produce higher grain yield and NUE. Therefore, the objectives of this study were as follows: (i) to quantify the variation between tillers regarding yield formation at different plant densities and N levels; and (ii) to build a model to determine the optimal planting density and N application strategies for realizing a high-yielding and high N efficient population structure.

## Materials and Methods

### Plant materials and growth conditions

The field experiments conducted in this study were carried out over two winter wheat growing seasons (2013–2014 and 2014–2015), at the experimental station of Shandong Agricultural University, Tai’an, China (36°09′N, 117°09′E, 128 m above sea level). This region has a warm and semi-humid continental monsoon climate, with an average total annual solar irradiance of 5.08 × 10^6^ kJ cm^−2^, an average annual temperature of 13.7 °C, and an average annual rainfall of 631.5 mm. The rainfall and mean temperature distributions of winter wheat growing stages are shown in Fig. S1. The soil in the study area is classified as a Eutric Cambisol according to the World Reference Base for Soil Resources (2014). The top 30 cm of the soil contained 14.7 g kg^−1^ organic matter, 1.24 g kg^−1^ total N, 87.2 mg  kg^−1^ available N, 9.6 mg kg^−1^ available P, and 85.3 mg kg^−1^ available K. The winter wheat cultivar used for the purposes of this study was Jimai 22 (JM22), which was grown in the experimental plots. Seeds were sown on October 10, 2013 and October 8, 2014, and plants were respectively harvested on June 9, 2014 and June 8, 2015. Disease, pests, and weeds in each treatment were well controlled by managers.

### Treatments and experimental design

The experiments were laid out in a two-factor completely randomized design with three replicates. The main plots were assigned to three plant densities (75 × 10^4^ plants ha^−1^, D_1_; 300 × 10^4^ plants ha^−1^, D_2_; and 525 × 10^4^ plants ha^−1^, D_3_). Subplots were assigned to three urea (N) fertilizer application rates (120 kg N ha^−1^, N_1_; 240 kg N ha^−1^, N_2_; and 360 kg N ha^−1^, N_3_). Each replicate plot (3 m × 3 m) consisted of 10 rows of wheat and two ridges. Row spacing, the width of rides, and a diagram of plant densities are shown in Fig. S2. Half the amount of N, 75 kg ha^−1^ P_2_O_5_, and 150 kg ha^−1^ K_2_O were mixed into the soil before planting the wheat. The remaining amount of N was applied at the jointing stage in each treatment.

### Observations of wheat population dynamics

Uniform plants in a 1 m^2^ area per plot were selected and tagged with labels for observations of population dynamics at the following stages: three-leaf, over-wintering, stem elongation, heading time (50% of plants headed), and maturity. Twenty plants were randomly selected and tagged with labels in each treatment for observation of tiller number at over-wintering, stem elongation, heading time, and maturity. The main stem was denoted as 0; primary tillers on the main stem in emergence order were referred to as I, II, III, IV, and V. Secondary tillers on the primary tillers in emergence order were referred to as I_1_ and II_1_ (Fig. S3). The effective tiller rate (ETR) was calculated using the equation: ETR = the number of tiller spikes per plant at maturity/the maximal number of tillers per plant ×100%. The ineffective tiller rate (ITR) = 100–ETR.

### Assessment of the grain-filling process

From 4 days after anthesis (DAA), 30 plants from each treatment in the field experiments were sampled at 4-day intervals until 36 DAA. Spikes were dried at 70 °C to constant weight, dehulled, and weighed. These data were used to characterize the grain-filling process using Richards’ growth equation, as described by Yang et al. (2004):

(1) \begin{eqnarray*}W=A/(1+B{e}^{-kt})^{1/N}.\end{eqnarray*}

Grain filling rate (G) was calculated as the derivative of Eq. (1):

(2) \begin{eqnarray*}G=AKB{e}^{-kt}/N(1+B{e}^{-kt})^{(N+1)/N}.\end{eqnarray*}

Integration of Eq. (2) gave the mean grain-filling rate (Eq. (3)) and the maximum grain-filling rate (Eq. (4)):

(3) \begin{eqnarray*}& & {G}_{\mathrm{mean}}=Ak/(2N+4)\end{eqnarray*}

(4) \begin{eqnarray*}& & {G}_{\mathrm{max}}=Ak(1+N)^{-(N+1)/N}\end{eqnarray*}

where *W* is the kernel weight (*g*), *A* is the final kernel weight (*g*), *t* is the time after anthesis (*d*), and *B*, *k*, and *N* are the coefficients determined by regression. The active grain-filling period (*T*) was defined as the period during which *W* constituted from 5% (*t*_1_) to 95% (*t*_2_) of *A*.

### Assessment of first internode microstructure

The microstructure of the first internode was assessed according to the methods described by Zheng et al. (2017). Fifteen days after anthesis, three plants from each treatment were sampled for microstructural observations. The middle section of the first internode (2 cm in length) of plants from each treatment was fixed in formalin:acetic acid:ethanol for 24 h, dehydrated with ethanol, and embedded in paraffin. Approximately 4-µm-thick sections were obtained using a microtome (Leica, Wetzlar, Germany), and these were sequentially stained with 1% safranin and 0.5% fast green. The stained cross-sections were observed and photographed using a Nikon DS-V3 microscope (Nikon, Tokyo, Japan). The number and area of vascular bundles at the internode were measured and calculated using Image-Pro Plus (Version 6.0; Media Cybernetics, Rockville, MD, USA).

### Measurements of single-stem biological yield and grain yield and its components

At maturity, the 20 tagged plants in each treatment were selected and divided into different spikes according to tillering order. The grains from individual spikes were oven dried at 70 °C to constant weight for determinations of grain number per spike and yield per spike. Euclidean distances were used to identify dissimilarities between the different tillers based on grain number and yield per spike.

Spikes in an area of 1 m^2^ from which no spikes had been sampled were harvested by hand to determine grain yield, number of kernels per spike, and 1,000-grain weight. Each measurement was performed on plants from three different plots.

### Polynomial regression with response surface analysis and evaluation of nitrogen-use efficiency

Trend surface simulation was used to analyze the effect of plant densities and N application rates on grain yield. The quadratic polynomial trend surface equation is as follows:

(5) \begin{eqnarray*}z={a}_{0}+{a}_{1}x+{a}_{2}y+{a}_{3}{x}^{\text{2}}+{a}_{4}xy+{a}_{5}{y}^{\text{2}}\end{eqnarray*}

where *z* is the grain yield (kg ha^−1^), *x* is plant density (plant ha^−1^), *y* is the N application rate (kg N ha^−1^), and a_0_, a_1_, a_2_, a_3_, a_4_ and a_5_ are the coefficients determined by regression. These coefficients were calculated by the least square method.

The partial factor productivity for the applied N (PFP_N_) was used to indicate NUE. PFP_N_ was calculated using the following equation:

(6) \begin{eqnarray*}PF{P}_{\mathrm{N}}={Y}_{\mathrm{N}}/N\end{eqnarray*}

where Y_N_ is grain yield in the N-applied plot and N is the nitrogen application rate.

### Statistical analysis and processing

Statistical analyses were carried out using Data Processing System software version 7.05 (DPS, Hangzhou, China). A multivariate ANOVA was conducted to determine the mean squares, degrees of freedom, and significance levels. Means were compared using LSD test and differences were considered significant at *P* < 0.05. The data for changes in the wheat population, effective tiller rate, grain number and yield per spike and the partial factor productivity for the applied N were averaged from the 2 years of the study. The trend surface simulation was analyzed using SPSS Statistics 24. Graphs were plotted using SigmaPlot 10.

## Results

### Results of variance analysis of the year (Yr), plant density (*D*), nitrogen application rate (*N*), and their interactions

Variance analysis was conducted using DPS7.05 to find the mean squares and significance (Tables 1 and 2). Gain yield (GY), spike number per m^2^ (SN), 100-grain weight (TGW), the effective tiller rate (ETR), partial factor productivity of applied N (PFP), yield per spike, grain number per spike, the maximum grain-filling rate (Gmax); the mean grain-filling rate (Gmean), the active grain-filling period (T), and the associated vascular bundle parameters showed no significant effects of year (Yr), the interaction between Yr, plant density (D) and nitrogen application rate (N). In contrast, GY, SN, TGW, ETR, PFP, and the associated grain-filling parameters were significantly influenced by D, N and D × N interactions (Tables 3 and 4).

**Table 1:** Analysis of variance of the effects of year (Yr), plant density (D), nitrogen application rate (N), and their interactions on the associated yield components parameters.

Source of variation	GY		SN		TGW		ETR		PFP		WP		Yield per spike		Grain number per spike	
	MS	*P*	MS	*P*	MS	*P*	MS	*P*	MS	*P*	MS	*P*	MS	*P*	MS	*P*
Year (Yr)	159,740	0.09	131	0.72	1.35	0.12	0.27	0.79	3.76	0.13	634	0.51	0.012	0.24	0.09	0.87
Yr × D	62,212	0.32	2,013	0.15	795	0.60	15,996	0.92	0.74	0.62	7,171	0.13	0.0097	0.32	1.90	0.54
Yr × N	5,353	0.91	16.17	0.98	101	0.87	231	0.90	0.25	0.85	563	0.68	0.0009	0.90	4.07	0.27
Yr × D × N	20,075	0.83	353	0.84	0.29	0.77	0.30	0.99	0.19	0.97	1,262	0.49	0.0055	0.63	2.51	0.52

**Notes.**

Ms mean square GY grain yield SN spike number per m^2^ TGW 100-grain weight ETR the effective tiller rate PFP partial factor productivity of applied *N* WP the wheat population at the jointing stage

The *P* value <0.05 was considered significant.

**Table 2:** Analysis of variance of the effects of year (Yr), plant density (*D*), nitrogen application rate (*N*), and their interactions on the associated grain-filling parameters.

Tiller group	Source of variation	Gmax		Gmean		T		NBVB		NSVB		NTVB		ABVB		ASVB	
		MS	*P*	MS	*P*	MS	*P*	MS	*P*	MS	*P*	MS	*P*	MS	*P*	MS	*P*
Superior	Year (Yr)	0.0001	0.81	0.0001	0.69	0.03	0.24	2.24	0.07	1.5	0.18	1.85	0.19	1,011,412	0.06	21,848	0.82
	Yr × D	0.0002	0.41	0.0004	0.63	0.04	0.22	0.13	0.82	0.0001	0.99	0.52	0.61	633	0.99	206,240	0.61
	Yr × N	0.0001	0.94	0.0001	0.92	0.01	0.77	0.07	0.89	0.06	0.93	0.80	0.47	203,118	0.47	89,503	0.80
	Yr × D × N	0.0001	0.68	0.0002	0.94	0.03	0.27	0.05	0.99	0.64	0.54	0.38	0.83	152,063	0.68	147,978	0.83
Inferior	Year (Yr)	0.0009	0.12	0.0001	0.91	0.0002	0.95	0.07	0.72	1.85	3.85	1.19	0.14	794,760	0.22	147,585	0.14
	Yr × D	0.0006	0.19	0.0001	0.79	0.01	0.83	0.57	0.37	32.02	66.50	0.69	0.28	888,164	0.18	57,738	0.42
	Yr × N	0.0011	0.06	0.0002	0.26	0.02	0.67	0.13	0.79	130	270.50	0.07	0.87	277,122	0.58	11,855	0.83
	Yr × D × N	0.0002	0.72	0.0001	0.78	0.02	0.85	0.55	0.43	0.02	0.04	0.07	0.97	54,529	0.98	14,425	0.93

**Notes.**

Ms mean square D plant density N nitrogen application rate Gmax the maximum grain-filling rate Gmean the mean grain-filling rate T the active grain-filling period NBVB the number of big vascular bundles NSVB the number of small vascular bundle NTVB the number of total vascular bundle ABVB the area of big vascular bundle ASVB the area of small vascular bundle

The *P* value < 0.05 was considered significant.

**Table 3:** The split plot ANOVA table for the associated yield components parameters.

Year	Source of variation	GY	SN	TGW	ETR	PFP	WP	Yield per spike	Grain number per spike
2013–2014	Block	93,558	30	0.04	29.52	1.86	218	0.01	3.06
	D	3,015,939^**^	3,084,323^**^	44.65^**^	7,994.75^**^	89.51^**^	440,7628^**^	7.04^**^	1,419^**^
	D Error	24,002	1,138	0.04	0.16	1.63	442	0.01	2.94
	N	23,218,384^**^	15,850^**^	13.54^**^	122.94^**^	2,876.23^**^	869,123^**^	0.20^**^	201^**^
	D × N	412,059^*^	6,453^**^	0.28^*^	6.67^**^	21.05^**^	65,927^**^	0.03^*^	11.34^*^
	N Error	111,701	726	0.08	0.54	2.96	1,140	0.01	4.18
2014–2015	Block	123,787	544	0.08	15.64	3.54	934	0.01	3.53
	D	4,998,595^**^	250,523^**^	39.66^**^	8,001.76^**^	70.96^**^	4,674,104^**^	7.79^**^	1,306^**^
	D Error	23,059	3,814	0.03	1.42	0.09	2,789	0.01	1.61
	N	47,088,641^**^	15,844^**^	14.10^**^	109.14^**^	2,946.75^**^	898,100	0.19^**^	183^**^
	D × N	709,291^**^	3,957^**^	0.24^*^	5.75^*^	21.88^**^	67,617^**^	0.05^**^	6.81^*^
	N Error	48,952	510	0.13	2.70	0.17	1,932	0.01	2.46

**Notes.**

D plant density N nitrogen application rate GY grain yield SN spike number per m^2^ TGW 100-grain weight ETR the effective tiller rate PFP partial factor productivity of applied *N* WP the wheat population at the jointing stage

Data represent mean square. ** and * represent significance at the 0.01 and 0.05 probability level, respectively.

**Table 4:** The split plot ANOVA table for the associated grain-filling parameters and vascular bundle parameters.

Year	Source of variation	Gmax		Gmean		T		NBVB		NSVB		NTVB		ABVB		ASVB	
		S	I	S	I	S	I	S	I	S	I	S	I	S	I	S	I
2013–2014	Block	0.0002	0.0001	0.0001	0.0001	0.0131	0.0003	0.04	1.81	0.15	1.44	0.26	0.70	211,898	516,335	496,285	153,037
	D	0.14^**^	0.14^**^	0.02^**^	0.05^**^	5.08^**^	7.47^**^	79.59^**^	21.92^**^	121.59^**^	16.78^**^	388.59^**^	73.37^**^	66,419,449^**^	43,232,960^**^	34,199,792^**^	5,948,765^**^
	D Error	0.0001	0.0003	0.0005	0.0001	0.0348	0.0329	1.37	0.31	0.54	0.39	1.93	1.20	156,576	754,001	360,743	14,672
	N	0.22^**^	0.178^**^	0.0523^**^	0.0709^**^	6.753^**^	18.92^**^	61.81^**^	30.04^**^	51.81^**^	65.33^**^	226.81^**^	182.26^**^	75,444,130^**^	40,140,666^**^	19,331,029^**^	3,226,157^**^
	D × N	0.01^**^	0.012^**^	0.01^**^	0.004^**^	2.22^**^	2.14^**^	1.48^*^	2.20^**^	3.037^*^	2.78^**^	4.31^*^	7.09^**^	771,158^*^	5,622,231^**^	1,929,184^*^	621,198^**^
	N Error	0.0004	0.0007	0.0005	0.0002	0.02	0.04	0.26	0.20	0.80	0.41	0.81	0.54	200,765	725,536	513,907	78,317
2014–2015	Block	0.0001	0.0001	0.0004	0.0001	0.05	0.0001	0.33	1.93	0.15	0.15	0.11	0.11	339,298	15,082	808,789	16,844
	D	0.15^**^	0.14^**^	0.03^*^	0.05^**^	6.37^**^	6.75^**^	70.78^**^	15.59^**^	121.59^**^	15.26^**^	379.11^**^	94.78^**^	66,952,005^**^	53,395,009^**^	34,406,001^**^	5,891,122^**^
	D Error	0.0002	0.0004	0.002	0.0002	0.0105	0.1062	2.61	0.37	0.537	0.59	1.56	0.22	408,608	736,204	375,627	11,798
	N	0.22^**^	0.19^**^	0.05^**^	0.07^**^	6.21^**^	20.58^**^	57^**^	29.15^**^	51.81^**^	64.93^**^	236.78^**^	180.11^**^	80,329,210^**^	34,688,599^**^	18,879,826^**^	3,751,582^**^
	D × N	0.01^**^	0.01^**^	0.01^*^	0.004^**^	2.87^**^	1.94^**^	1.61^*^	7.43^**^	3.04^*^	1.87^*^	3.56^*^	8.06^**^	1,116,807^*^	6,940,629^**^	3,431,991^**^	637,779^**^
	N Error	0.0002	0.0001	0.001	0.0001	0.02	0.07	0.30	0.61	0.80	0.44	1.02	0.41	307,715	190,248	251,547	80,451

**Notes.**

D plant density N nitrogen application rate Gmax the maximum grain-filling rate Gmean the mean grain-filling rate T the active grain-filling period NBVB the number of big vascular bundles NSVB the number of small vascular bundle NTVB the number of total vascular bundle ABVB the area of big vascular bundle ASVB the area of small vascular bundle S superior tiller group I inferior tiller group

Data represent mean square. ** and * represent significance at the 0.01 and 0.05 probability level, respectively.

### Grain yield and its components

Grain yield (GY), spike number per m^2^ (SN), grain number (GN), and 1,000-grain weight (TGW) were found to be significantly influenced by planting density and N application rate (Table 5). Although increasing plant density significantly enhanced GY and SN, both GN and TGW were significantly decreased at higher plant densities. As N application rate was increased from 120 to 240 kg ha^−1^, the 2-year average GY was significantly enhanced by 43.5%, 34.4%, and 35.2% under planting densities D1, D2, and D3, respectively. Compared with the D2N1 treatment, SN was significantly increased, by 5% and 12% in treatments D2N2 and D2N3, respectively. No significant differences in GY were observed between the N2 and N3 treatments across densities D1 and D2. However, compared with D3N2, the D3N3 treatment significantly decreased GY.

**Table 5:** Grain yield (GY), spike number (SN), grain number (GN), and 1000-grain weight (TGW) at different treatments.

Treatment	GY (kg ha^−1^)	SN (×10^4^ ha^−1^)	GN (No. spike^−1^)	TGW (g)
D1N1	6,999 d	641 fg	44.3 c	43.0 b
D1N2	10,041 b	628 g	47.4 b	44.6 a
D1N3	10,222 b	687 f	51.0 a	44.5 a
D2N1	8,249 c	935 d	37.5 ef	40.5 e
D2N2	11,083 a	984 bc	38.6 e	42.6 c
D2N3	11,054 a	1,046 a	39.9 d	42.4 c
D3N1	8,132 c	877 e	32.2 h	39.7 f
D3N2	10,994 a	1,025 ab	34.0 g	41.4 d
D3N3	10,127 b	960 cd	36.9 f	40.8 e

**Notes.**

Values followed by different letters within the columns are significantly different at the 0.05 probability level.

In addition, we found that that SN was only significantly positively correlated with GY (Table S1), whereas it was significantly negatively correlated with TGW and GN. Furthermore, we identified positive direct path coefficients for SN and TGW to GY, respectively, whereas the indirect path coefficients were negative for SN and TGW to GY (Tables S2 and S3).

### Changes in the wheat population and effective tiller rate

**Figure 1:**
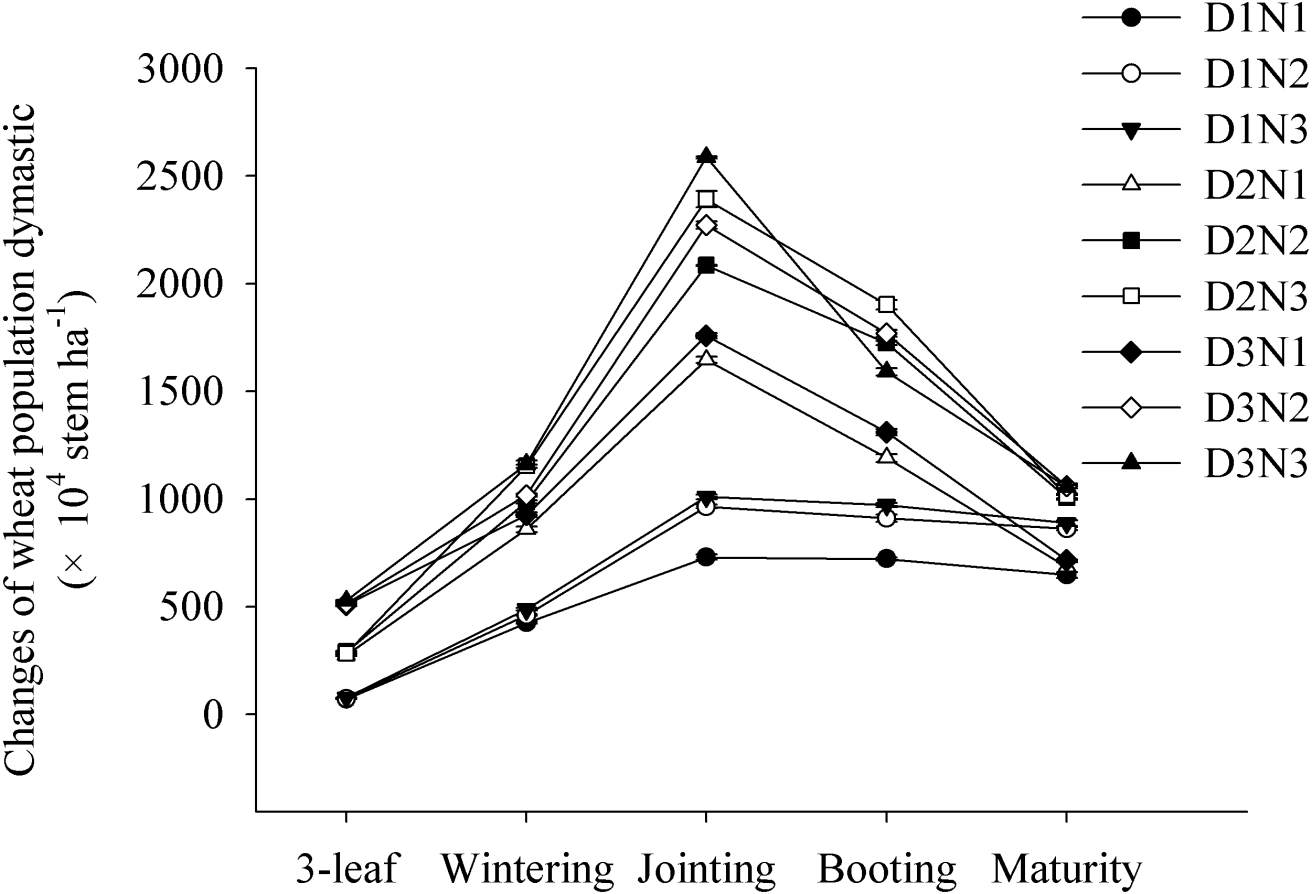
Effects of plant densities and application of nitrogen rates on the changes of stems of winter wheat. Symbols represent means ± standard error. Vertical bars indicate standard error.

We found that the wheat populations rapidly increased from the three-leaf stage to the jointing stage, reaching a maximum before subsequently decreasing. Wheat populations increased significantly with increasing planting density and N application rate. At the jointing stage, compared with D1N1 treatment, the populations in treatments D2N1 and D3N1 increased by 125% and 141%, respectively. Similarly, compared with the D2N1 treatment, the populations obtained in the D2N2 and D2N3 treatments increased by 26.7% and 45.4%, respectively (Fig. 1).

**Figure 2:**
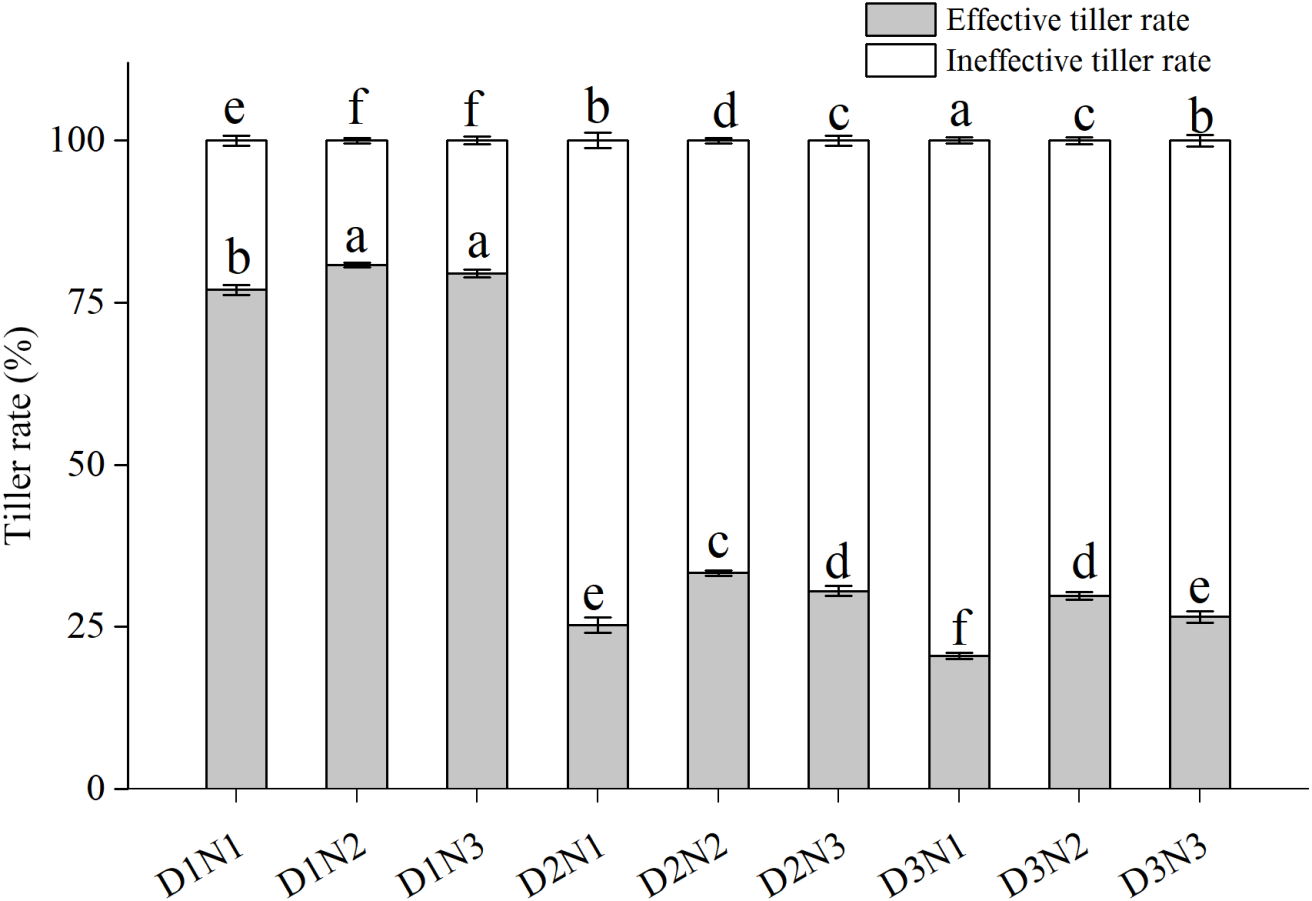
Effects of plant densities and application of nitrogen rates on the effective tiller rate and ineffective tiller rate. Symbols represent means ± standard error. Vertical bars indicate standard error. Different letters within the columns are significantly different at the 0.05 probability level.

Effective tiller rate (ETR) tended to be higher than ineffective tiller rate (ITR) in the low plant density treatments (D1) (Fig. 2). ITR increased significantly with increases in plant density, with that in treatments D2N1 and D3N1 being more than doubling in both growing seasons compared with D1N1. Although application of N significantly increased ETR (*P* < 0.05), with that of D2N2 increasing by 24.1% compared with D2N1, the increase was less pronounced (20.8%) at the highest N application level (N3), and in both growing seasons, ETR in the D2N3 treatment was lower than that in the D2N2 treatment.

### Grain number and yield per spike of different tillers

**Figure 3:**
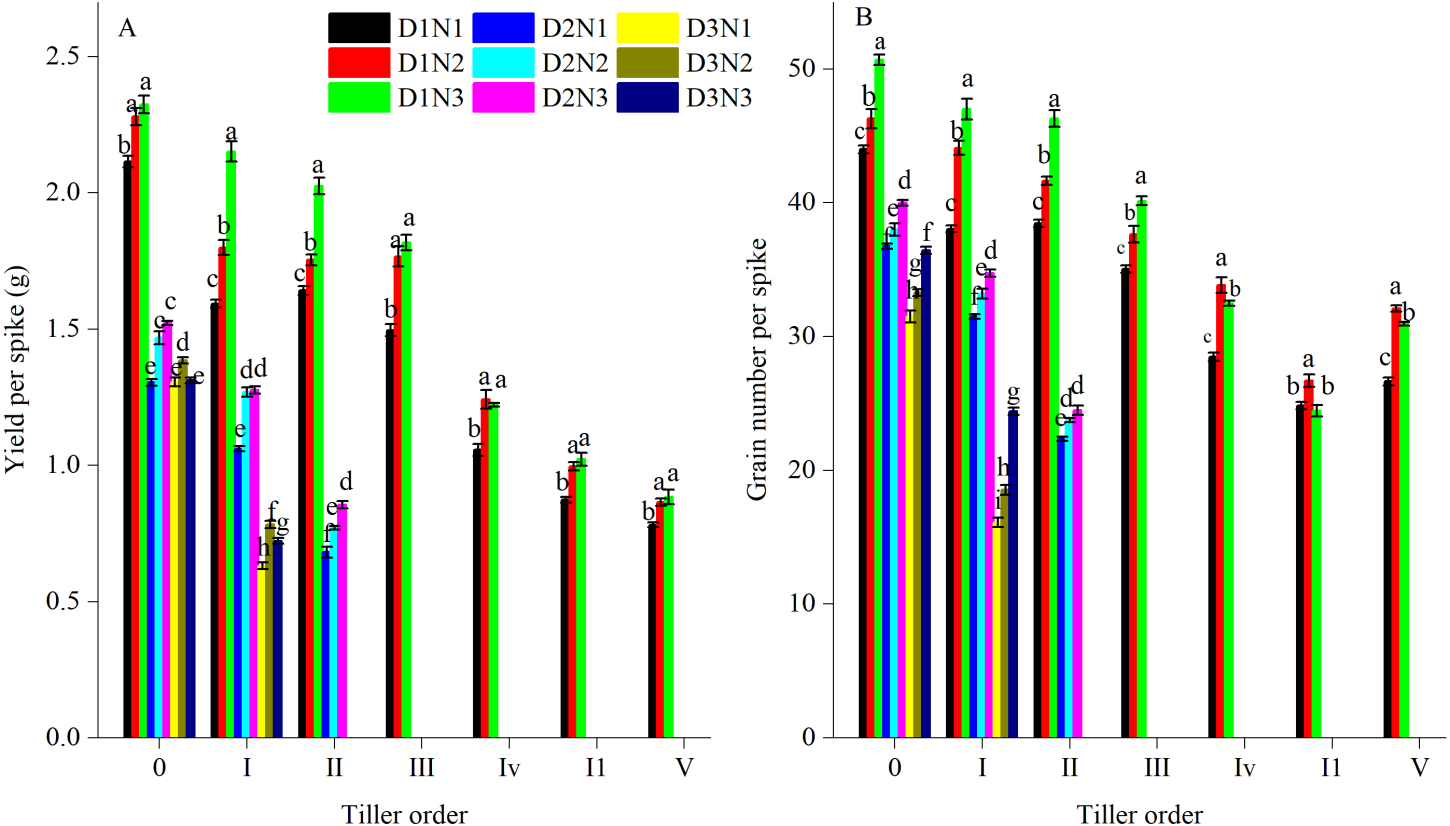
Effects of plant densities and application of nitrogen rates on yield per spike (A) and grain number per spike (B) of different tillers. Different letters within the columns are significantly different at the 0.05 probability level.

Grain number and yield per spike appeared to vary among different tiller orders (Fig. 3). The main stem had the highest GN and yield per spike. With the sequence of tiller emergence, GN and yield per spike of tillers gradually declined. For all tillers, increasing plant density resulted in a decrease in GN and yield per spike. For example, compared with treatment D1N2, yield per spike of I order tillers for D2N2 and D3N2 decreased by 29.5% and 56.5%, respectively. An increase in N application resulted in an increase in GN and yield per spike. In comparison with treatment D1N1, the yield per spike of order 0, I, and II tillers in response to treatment D1N2 was 7.8%, 13.0%, and 6.9%, respectively (Fig. 3A). Compared with the D1N2 treatment, the GN per spike of order I tillers for D2N2 and D3N2 decreased by 24.7% and 57.9%, respectively, whereas compared with the D1N1 treatment, the GN of order 0, I, and II tillers increased by 5.2%, 15.9%, and 8.3%, respectively, when N was applied at 240 kg N ha^−1^ (Fig. 3B). In addition, with an increase in plant density, there was a decrease in the number of fertile tillers. Under higher plant densities, only order I and II tillers were ear-bearing, and in the D3 treatment, only one order I tiller produced ears.

### Grain-filling process and microstructure of the first internode of superior and inferior tiller groups

**Figure 4:**
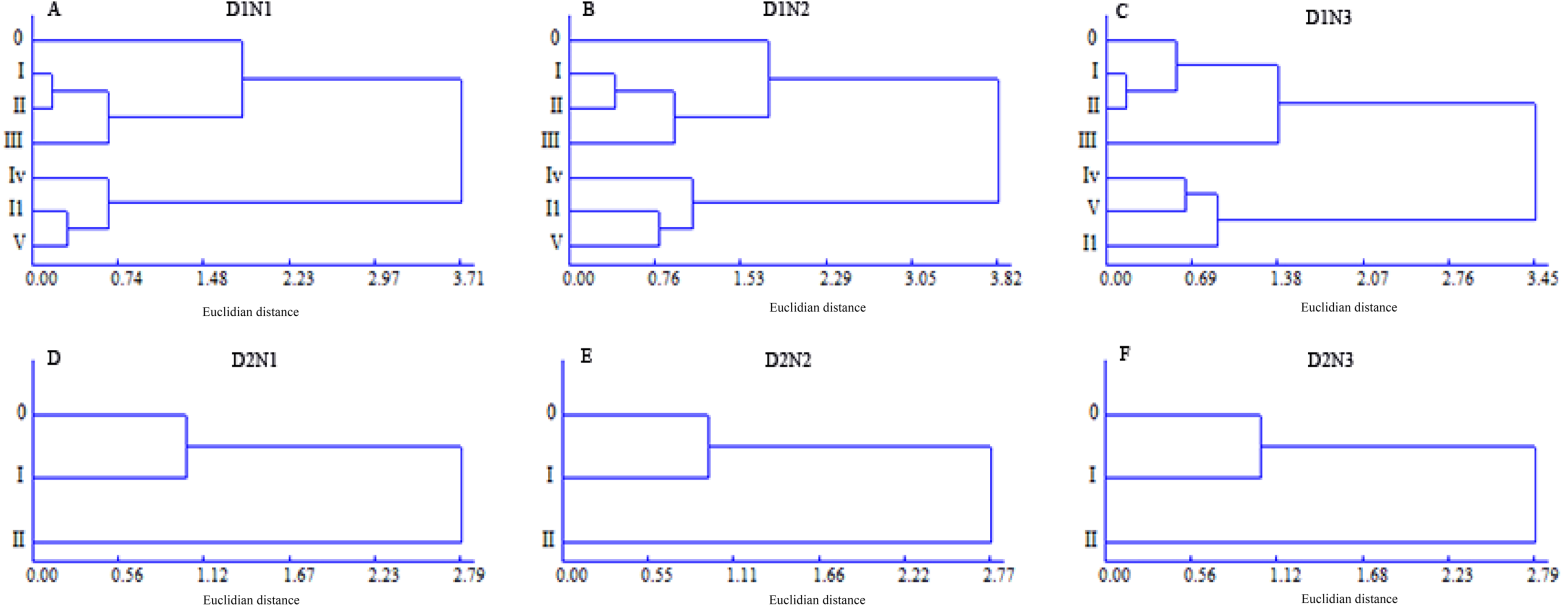
Dendrogram of the classification of different wheat tillers based on grain number and yield per spike.

The main stem and tillers in density D1 plants were categorized into two groups, and defined as the superior tiller group, including order 0, I, II, and III tillers, and the inferior tiller group, including order IV, I1, and V tillers, whereas the superior tiller group for the density D2 plants included order 0 and I tillers and the inferior tiller group included order II tillers (Fig. 4). Plants at a density of D3 had just a single tiller; however, dissimilarities between GN and yield per spike meant that a distinction could be made between the main stem, as the superior group, and tiller I as the inferior tiller group. The grain-filling processes and the microstructure of the first internode of the two tiller groups were then investigated to identify the regulatory mechanisms whereby plant density and N application influence yield formation.

The maximum grain-filling rate (Gmax) and the mean grain-filling rate (Gmean) of superior and inferior tiller groups decreased with an increase in plant density, but were enhanced by increasing N application (Table 6). For example, compared with treatment D2N1, the Gmax of the superior tiller group increased by 17.6% and 15.7% for treatments D2N2 and D2N3, respectively, whereas compared with treatment D1N1, the Gmean of the inferior tiller group increased by 15.0% and 19.2% for D1N2 and D1N3, respectively. Furthermore, we found that the active grain-filling period (T) of the inferior tiller group increased with increasing N application.

**Table 6:** The effects of plant density and nitrogen application on grain-filling parameters and vascular bundles.

Tiller group	Treatment	Gmax (mg per kernel d^−1^)	Gmean (mg per kernel d^−1^)	T (d)	NBVB	NSVB	NTVB	ABVB (*μ*m^2^)	ASVB (*μ*m^2^)
Superior	D1N1	2.27 f	1.47 de	30.3 e	23.5 d	29.2 c	52.7 c	8,081.3 cd	5,344.4 d
	D1N2	2.56 a	1.52 bc	29.6 f	25.7 c	32.5 a	58.2 b	14,001.2 a	9,279.7.1 a
	D1N3	2.50 b	1.58 a	31.8 c	29.0 a	33.2 a	62.2 a	14,128.2 a	8,479.3 b
	D2N1	2.04 h	1.33 f	32.8 a	21.2 f	24.5 e	45.7 e	5,107.8 e	3,891.6 e
	D2N2	2.40 c	1.54 b	30.7 d	25.2 c	27.0 d	52.2 c	10151.3 b	5,709.3 d
	D2N3	2.36 d	1.50 cd	32.7 a	27.0 b	31.0 b	58.0 b	10113.8 b	7,491.3 c
	D3N1	2.06 h	1.33 f	30.6 d	18.2 g	22.3 f	40.5 f	3,899.9 f	2,961.8 f
	D3N2	2.25 g	1.46 e	32.1 b	21.0 f	24.5 e	45.5 e	7,844.2 d	4,163.3 e
	D3N3	2.29 e	1.46 e	32.8 a	22.3 e	26.0 d	48.3 d	8,466.4 c	4,279.7 e
Inferior	D1N1	1.82 f	1.20 e	32.2 e	18.5 b	23.2 e	41.7 e	6,022.6 d	1,369.8 d
	D1N2	2.11 b	1.38 b	32.9 d	19.7 a	26.3 bc	46.0 b	10,126.3 b	2,891.9 b
	D1N3	2.19 a	1.43 a	33.8 c	20.3 a	28.2 a	49.0 a	13,375.6 a	3,526.1 a
	D2N1	1.74 g	1.18 e	31.8 f	16.2 d	21.5 f	37.7 g	4,136.1 f	1,133.6 d
	D2N2	1.89 e	1.27 d	35.4 b	19.8 a	24.3 d	44.1 d	6,061.5 d	2,023.3 c
	D2N3	1.99 c	1.29 c	35.9 a	19.5 a	25.3 c	44.8 c	6,735.1 cd	2,114.2 c
	D3N1	1.71 h	1.10 f	32.8 d	13.5 e	19.5 g	33.0 h	4,960.3 e	710.2 e
	D3N2	1.77 g	1.19 e	35.5 b	17.3 c	23.7 de	41.0 f	6,152.5 d	1,128.8 d
	D3N3	1.94 d	1.27 d	35.4 b	18.5 b	26.7 b	45.2 c	7,146.4 c	1,095.7 d

**Notes.**

Gmax the maximum grain-filling rate Gmean the mean grain-filling rate T the active grain-filling period NBVB the number of big vascular bundles NSVB the number of small vascular bundle NTVB the number of total vascular bundle ABVB the area of big vascular bundle ASVB the area of small vascular bundle

Values followed by different letters within the columns are significantly different at the 0.05 probability level.

On the basis of the results of cluster analysis, we examined the microstructures of the first internode in superior and inferior tiller groups. Our observations revealed that the number of large vascular bundles (NBVB), small vascular bundles (NSVB), and total number of vascular bundles (NTVB), as well as the area of the large vascular bundles (ABVB) and small vascular bundles (ASVB) of the superior tiller group, were all higher than those of the inferior tiller group (Table 6). An increase in plant density significantly decreased NBVB, NSVB, NTVB, ABVB, and ASVB, whereas an increase in N application significantly increased NBVB, NSVB, NTVB, ABVB, and ASVB. For example, compared with treatment D2N1, the NTVB of the superior and inferior tiller groups in the D2N2 treatment increased by 6.5 and 6.4 per culm, respectively. Compared with the D2N1 treatment, the ABVB of the superior tiller group increased significantly by 99% and 98% in the D2N2 and D2N3 treatments, and that of the inferior tiller group increased by 46.6% and 62.8%, respectively.

### Relationships between individual yield per spike and vascular bundles

**Figure 5:**
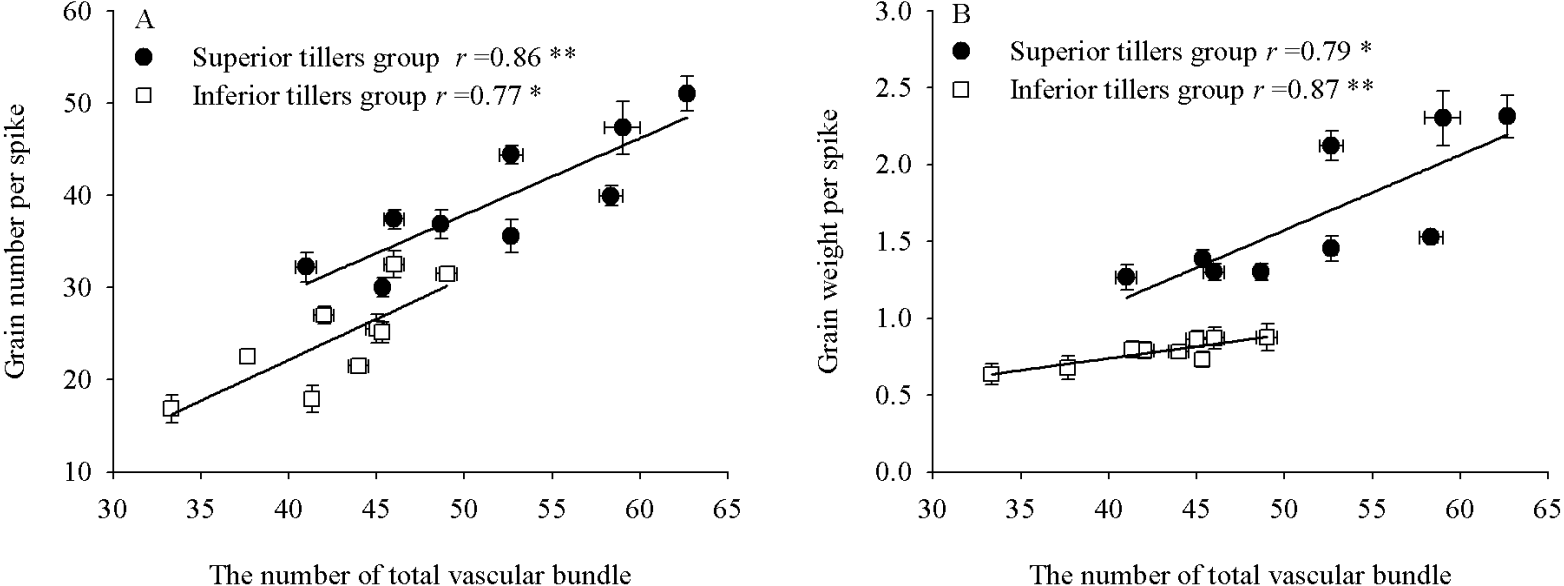
The relationship between single grain yield and the total number vascular bundle of superior and inferior tiller groups.

We observed significant correlations between individual yield and vascular bundles (Fig. 5). The grain number and yield per spike of the superior tiller group were significantly positively correlated with the NTVB (*r* = 0.86∗∗, *P* <0.01; *r* = 0.79*, *P* <0.05, respectively), as were the grain number and yield per spike of the inferior tiller group (*r* = 0.77 *, *P* <0.05; *r* = 0.87 **, *P* <0.01, respectively). Significant correlations were likewise observed between vascular bundle areas and grain-filling rates (Table 7).

**Table 7:** The relationship between the vascular bundle area and the grain-filling rate of superior and inferior tiller groups.

Tiller group	Vascular bundle area	Gmax	Gmean
Superior tiller group	ABVB	0.97^**^	0.89^**^
	ASVB	0.91^**^	0.80^**^
Inferior tiller group	ABVB	0.95^**^	0.93^**^
	ASVB	0.93^**^	0.94^**^

**Notes.**

Gmax the maximum grain-filling rate Gmean the mean grain-filling rate ABVB the area of big vascular bundle ASVB the area of small vascular bundle

Correlation coefficients (*r*) are calculated and asterisks (**) represent significance at the 0.01 probability level.

### Trend surface analysis and evaluation of nitrogen-use efficiency

**Figure 6:**
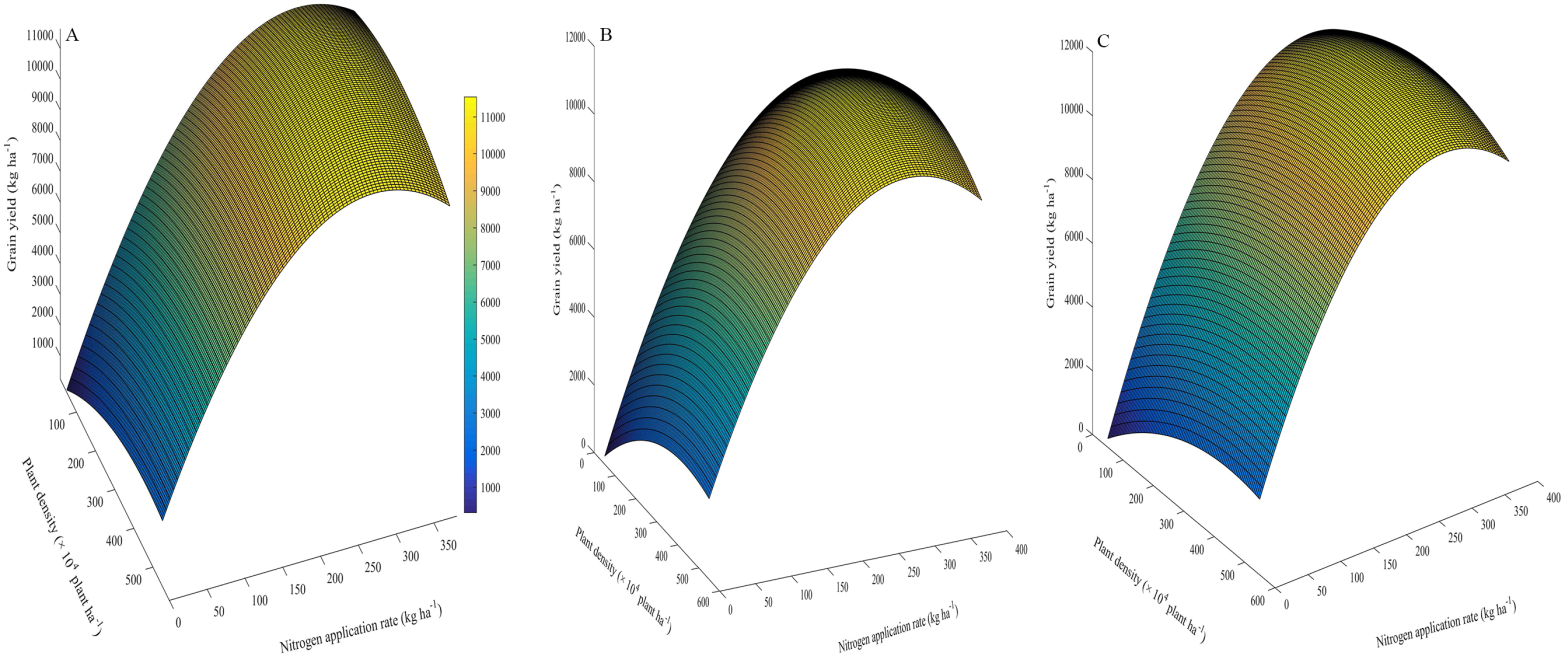
3D graphic surface optimization of grain yield versus plant density and nitrogen application rate (A, 2013–2014; B, 2014–2015; C, the average of the two years).

The effects of plant density and N application on GY are shown by the coefficients of second order polynomials (Fig. 6). These coefficients were calculated by the least square method, as given in the equations (Table 8). For example, the total determination coefficient *R*^2^ = 0.992 implies that variations of 99.2% for GY are attributable to plant density and *N* application rate. If the partial derivative of the equation is zero, two equations can be constructed as follows: 13.9 − 0.012*y* − 0.032*x* = 0 and 66 − 0.012*x* − 0.214*y* = 0, and 11.3 − 0.011*y* − 0.024*x* = 0 and 68 − 0.011*x* − 0.222*y* = 0, respectively. By solving the equation groups, the following results can be obtained: *x* = 326 (×10 ^4^plant ha^−1^), *y* = 290 (kg N ha^−1^) in the first growing season, and *x* = 338 (×10 ^4^ plant ha^−1^), *y* = 289 (kg N ha^−1^) in the second growing season, respectively. Similarly, the average results in two years can be obtained: *x* = 336 (×10 ^4^plant ha^−1^), *y* = 290 (kg N ha^−1^). Via inputting the data of these two variables into the equation, the maximum GY and its partial factor productivity of applied N could be calculated: *Z*_MaxGY_ = 11,524 (kg ha^−1^) and Max_GY_ PFP = 39.7 (kg kg^−1^).

**Table 8:** The quadratic polynomial trend surface equations and PFP.

Year	Equation	*R*^2^	*P*	*x*	*y*	*z*	PFP
2013–2014	*z*= 13.9*x*+ 66*y*= 0.012*xy*= 0.016*x*^2^= 0.107*y*^2^= 421	0.992	^**^	326	290	11,417	39.4
2014–2015	*z*= 11.3*x*+ 68*y*= 0.011*xy*= 0.012*x*^2^= 0.111*y*^2^= 219	0.996	^**^	338	289	11,438	39.6
Mean	*z*= 12.6*x*+ 67*y*= 0.011*xy*= 0.014*x*^2^= 0.109*y*^2^= 320	0.991	^**^	336	290	11,524	39.7

**Notes.**

*R*^2^ the coefficient of determination *z* represents the grain yield (kg ha^−1^) *x* represents plant density (plant ha^−1^) *y* represents the N application rate (kg N ha^−1^) PFP partial factor productivity of applied N (kg kg^−1^)

** represents significance at the 0.01 probability level.

**Figure 7:**
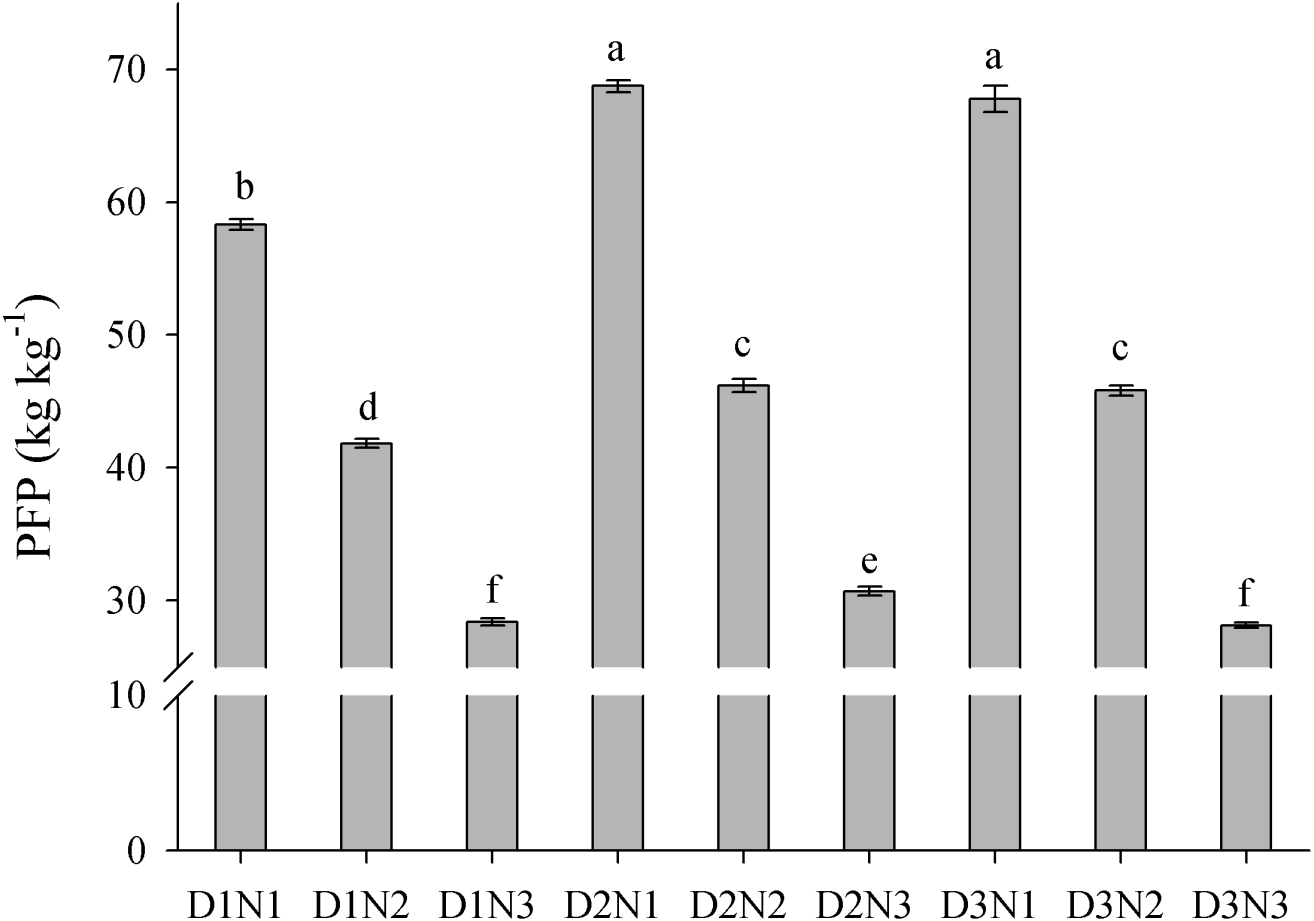
Effects of plant densities and application of nitrogen rates on the PFP. PFP represents the partial factor productivity of applied N. Different letters within the columns are significantly different at the 0.05 probability level.

**Figure 8:**
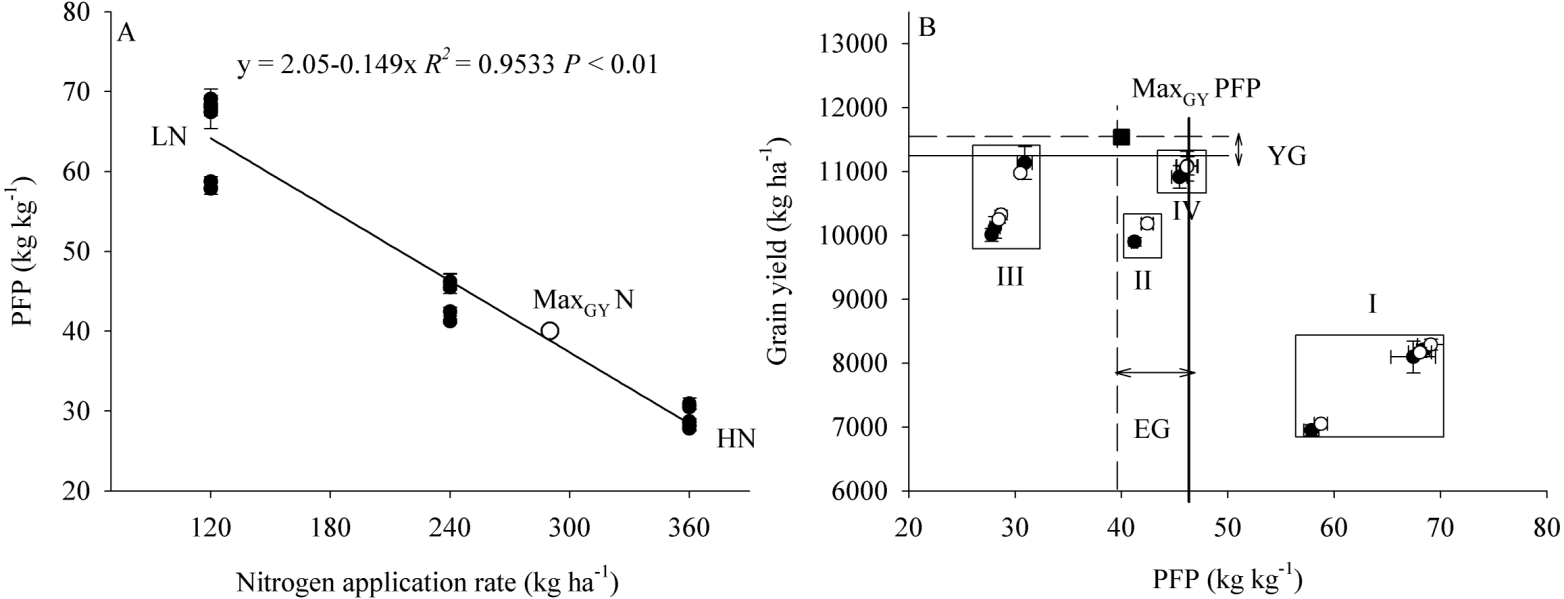
The relationship between PFP and nitrogen application rate and grain yield. PFP represents the partial factor productivity of applied *N*. Max_*GY*_*N* represents the nitrogen application rate, which achieve the maximum grain yield calculated by the second order polynomials trend surface. Max_*GY*_*PFP* represents the partial factor productivity of applied *N*, which achieve the maximum grain yield. YG represents yield gap between the maximum grain yield and D2N2 treatment. EG represents the PFP gap between the maximum grain yield and D2N2 treatment.

The results showed that an increase in plant density (D1 to D2) significantly increased PFP, but there was no significant difference in PFP between densities D2 and D3 (Fig. 7). In contrast, an increase in N application significantly decreased PFP. Compared with treatment D2N3, the average PFP_N_ in the 2 years of the study was increased by 50.4% in response to treatment D2N2. Furthermore, we found that PFP was significantly negatively correlated with the rate of N application (Fig. 8A). Taking GY and NUE into consideration, treatments could be classified into four groups: (I) lower GY with higher NUE (D1N1, D2N1, and D3N1), (II) high yield with high NUE (D1N2), (III) higher yield with lower NUE (D1N3, D2N3, and D3N3), and (IV) higher yield with high NUE (D2N2 and D3N2) (Fig. 8B). These results indicate that increasing plant density and decreasing N application could be a useful strategy for achieving high GY and NUE.

## Discussion

### Effects of plant density and nitrogen application on grain yield and its components

Yield variability is largely controlled by plant density and N fertilizer input (Sukumaran et al., 2015; Liu et al., 2016). In the current study, we found that plant density, N application rate, and their interactions significantly affected GY. Grain yield increased with an increase in plant density and N application, which can be explained in terms of an increase in SN, whereas, both GN and TGW were reduced by increasing plant density. Previously, Li et al. (2016b) observed that SN was significantly increased by an increase in plant density, resulting in higher grain yield, whereas TGW and GN were decreased, which is partially consistent with our results. Large changes in yield can only be determined by variation in the grain number per m^2^, which is primarily associated with SN (Slafer, Savin & Sadras, 2014). In the present study, path analysis revealed that SN appears to be the most important factor determining GY, thereby indicating that preferentially optimizing the SN is an important measure for increasing grain yield.

Spike number includes the number main stem and tiller spikes and is determined by seeding rate and tiller generation and survival (Lloveras et al., 2004; Dreccer et al., 2013). Tillers arise from tiller buds initiated from the axillary meristems in the axils of leaves on the main shoot and tiller shoots, and the development of wheat tillers is regulated by numerous factors. Regarding genetic characteristics, multi-tiller or inhibition genes have been shown to control the development of tillers (Xue et al., 2013; Hendriks et al., 2016). Environmental factors and agronomic management, such as planting density and N application, also affect the initiation and cessation of tillering (Evers et al., 2011; Huang et al., 2013). The results of the present study indicate that an increase in plant density increases the effective tiller rate and decreases the number of high-position tiller spikes. In contrast with the low plant density (D1) treatments, we found that only the main stem and order I and II tillers survive to constitute the final population under density D2 (300 plants m^−2^).

Plant density-regulated tiller development may be related mainly to the variation in light within the canopy structure. In previous studies, light interception has been shown to increase with an increase in plant density, leading to less photosynthetically active radiation penetrating to the lower vegetation layer (Mao et al., 2014; Xue et al., 2015). Given that the development of tillers is asynchronous, the low-position tillers develop earlier than the high-position tillers. The former occupy the upper space and preempt the uppermost light source, shading the late-emerging tillers (Wang et al., 2016a). The cessation of tillering is induced when the fraction of light intercepted by the canopy exceeds a threshold (Evers et al., 2006). Plant density affects not only light quantity but also light quality, and the ratio of red to far-red light at the base of the canopy is reduced by higher plant densities (Rondanini et al., 2017), resulting in the inhibition of bud growth (Holalu & Finlayson, 2017). In addition, leaf area and leaf number both decrease as the tiller position shifts from low to high (Lafarge, Broad & Hammer, 2002). These factors may contribute to decreases in the photosynthetic activity of leaves on the late emerging tillers, due to the lack of photosynthetically active radiation and photosynthetic area, thereby resulting in lower sucrose levels, which would suppress tiller bud outgrowth (Mason et al., 2014; Kebrom & Mullet, 2015).

Nitrogen is one of the essential nutrient elements for crop growth and yield formation. In the present study, we found that the number of stems per m^2^ was significantly increased by an increase in N application, and the effective tiller rate was also increased. Therefore, the final SN was increased by a high N supply (N2 and N3), compared with low N application (N1). N deficiency inhibits tiller bud elongation (Luo et al., 2017), whereas N application has been shown to promote tillering via the accumulation of microRNA393 (miR393) in response to upregulation of OsmiR393 (Li et al., 2016c). In addition to genetic factors, an interaction between N and certain hormones is known to play a role in tiller development (Domagalska & Leyser, 2011; Xu et al., 2015b). Nitrogen application promotes cytokinin biosynthesis, and inhibits its degradation, thereby inducing tiller bud development (Liu et al., 2011), and also affects auxin transport and strigolactone synthesis to regulate axillary bud activation (Jong et al., 2014). However, supra-optimal N application may result in the vigorous vegetative growth (Nasim et al., 2016; Ata-Ul-Karim et al., 2017). In the present study, we found that our highest N application rate (N3) decreased the effective tiller rate compared with N2 treatments, and, notably, a high N supply combined with a high plant density (D3N3) resulted in lodging (Fig. S4).

Plant density and N application have significant effects on culm development. Vascular bundle number (NVB) and area (AVB) are the major anatomical features influencing stem-breaking strength and transportation (Tian et al., 2015). We found that whereas increasing plant density significantly decreased NVB and AVB at the first internode, an increase in N application increased NVB and AVB, and GN and yield per spike also increased with excessive N application, Moreover, the results of correlation analysis indicated that the number vascular bundle was significantly positively correlated with yield per spike, and that vascular bundle area was significantly positively correlated with grain filling rate. These results indicate that an appropriate combination of increased planting density and N application could regulate culm quality to facilitate the transport of photoassimilates to the grain sink, resulting in an acceleration of the grain-filling rate and enhancement of yield per spike.

### Optimized plant density and nitrogen application to enhance grain yield and nitrogen-use efficiency

Nutrient competition, mainly with respect to N, occurs between tillers (Assuero et al., 2012), and is intensified by increased plant density. Therefore, it should be possible to optimize plant density and N application to regulate tiller development and spike number, thereby achieving high yield and high NUE via a trade-off between the three yield components. Cluster analysis showed that the low- and high-position tiller spikes can be classified into superior and inferior tiller groups, respectively. The yield per spike, grain-filling rate and NVB and AVB in the superior tiller group were all higher than those in the inferior tiller group. In the present study, we found that increasing plant density reduced the number of high-position tillers, and that under a high plant density (300 × 10^4^ plant ha^−1^) the spikes consisted of the superior group. Increasing plant density increased the partial factor productivity of applied N (PFP), because the density of roots in soil was increased by increasing plant density, which enhances N uptake (Dai et al., 2014). Although increasing N application increased GY, the PFP was significantly decreased. We found that PFP was significantly negatively correlated with the N application rate, which indicates that increasing NUE should properly reduce N application. Second order polynomial trend surface analysis revealed that a plant density of 336 × 10^4^ plants ha^−1^ and an N application rate of 290 kg N ha^−1^ could produce the maximum GY (Max_GY_). Although the yield gap between Max_GY_ and that obtained with treatment D2N2 was 453 kg ha^−1^, the corresponding PFP (39.7 kg kg^−1^) was obviously high than that for N application at 360 kg N ha^−1^.

## Conclusion

Wheat spikes were classified into superior (including the main stem and low-position tillers) and inferior (high-position tillers) groups. The superior groups had higher grain number and yield per spike owing to a larger number of vascular bundles and faster grain-filling rate. We proposed and verified a technical approach to achieve a higher grain yield and high plant nitrogen-use efficiency through optimizing plant density and N application to regulate tiller growth, which appropriately increasing plant densities (from 75 to 336 plants m^−2^) and reducing nitrogen application (from 360 to 290 kg N ha^−1^), the number of inferior tillers can be reduced, thereby optimizing superior tiller development to generate a rational population structure.

##  Supplemental Information

10.7717/peerj.6484/supp-1 Figure S1 Mean daily temperature and monthly precipitation in growing seasons of winter wheat in 2013–2014 and 2014–2015

10.7717/peerj.6484/supp-2 Figure S2 Diagram showing the planting of the experiment Each plot (3 × 3 m) consisted of 10 rows of wheat and two ridges (A). A schematic diagram showing 75 ×10^4^ (B), 300 ×10^4^ (C), and 525 ×10^4^ (D) plant ha^−^1 over two years.

10.7717/peerj.6484/supp-3 Figure S3 The diagram of the tiller emerging order The main stem was denoted as 0; primary tillers on the main stem in emergence order were referred to as I, II, III, IV, and V. Secondary tillers on the primary tillers in emergence order were referred to as I _1_ and II _1_.

10.7717/peerj.6484/supp-4 Figure S4 Lodging emergence at early dough stage under D3N3 treatment

10.7717/peerj.6484/supp-5 Table S1 Correlation analysis of grain yield and yield components GY, grain yield; SN, spike number; GN, grain number; GW grain weight. Correlation coefficients (*r*) are calculated and asterisks (∗∗) represent significance at the 0.01 probability level and asterisks (∗) represent significance at the 0.05 probability level.

10.7717/peerj.6484/supp-6 Table S2 Partial correlation analysis of grain yield and yield components GY, grain yield; SN, spike number; GN, grain number; GW grain weight. Correlation coefficients (*r*) are calculated and asterisks (∗∗) represent significance at the 0.01 probability level.

10.7717/peerj.6484/supp-7 Table S3 Path analysis of yield components and grain yield GY, grain yield; SN, spike number; GN, grain number; GW grain weight.

10.7717/peerj.6484/supp-8 Data S1 Raw data

## Additional Information and Declarations

### Data Availability

The following information was supplied regarding data availability: The raw data are available in Supplemental Files.
