# Supplementary figures and images for: Optimizing plant density and nitrogen application to manipulate tiller growth and increase grain yield and nitrogen-use efficiency in winter wheat
Source: PeerJ. 2019 Feb 26;7:e6484. doi: 10.7717/peerj.6484 (PMC6396748; doi:10.7717/peerj.6484)

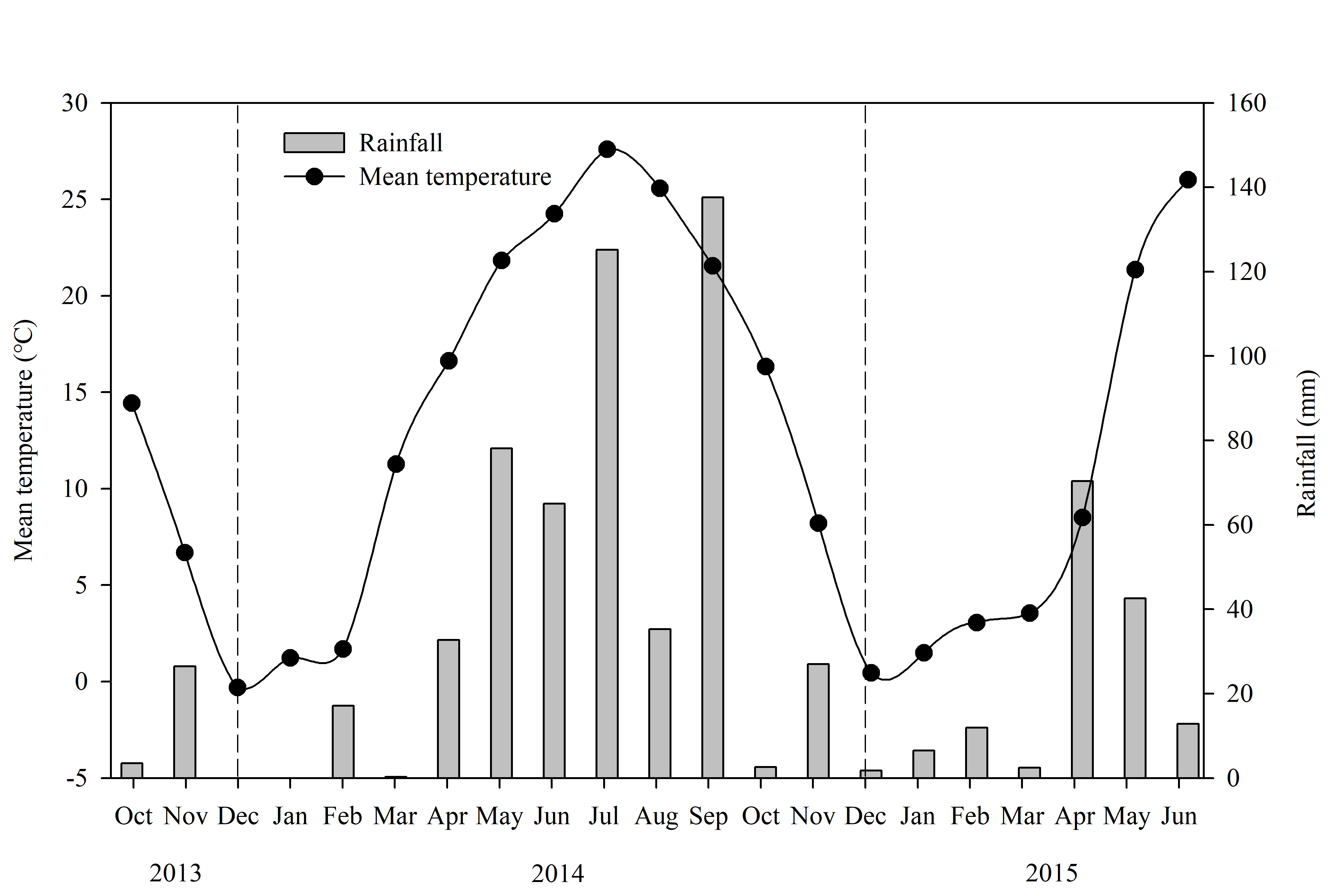

Supplement: Figure S1 [file peerj-07-6484-s001.png]

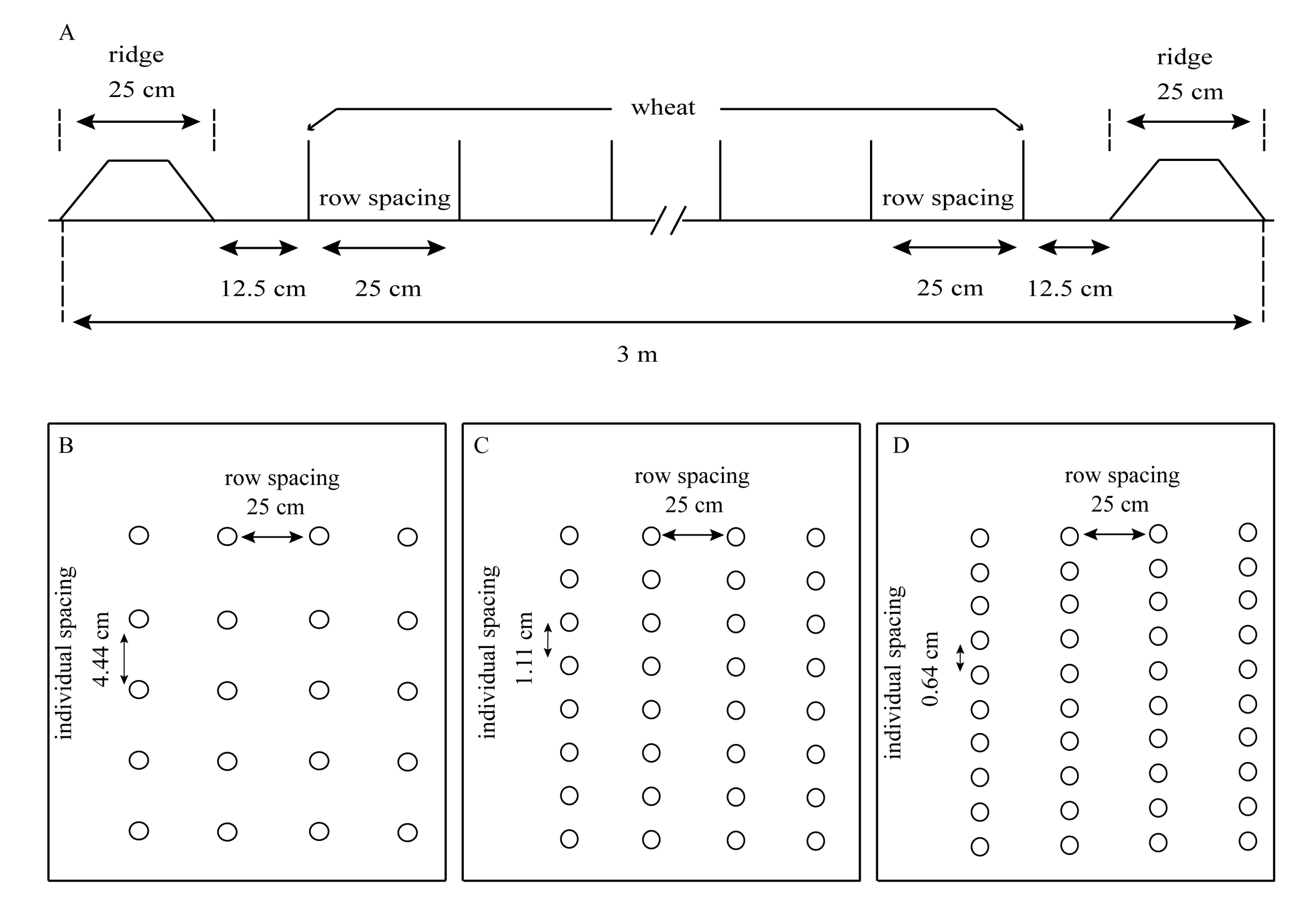

Supplement: Figure S2 — Each plot (3 × 3 m) consisted of 10 rows of wheat and two ridges (A). A schematic diagram showing 75 ×104 (B), 300 ×104 (C), and 525 ×104 (D) plant ha−1 over two years. [file peerj-07-6484-s002.png]

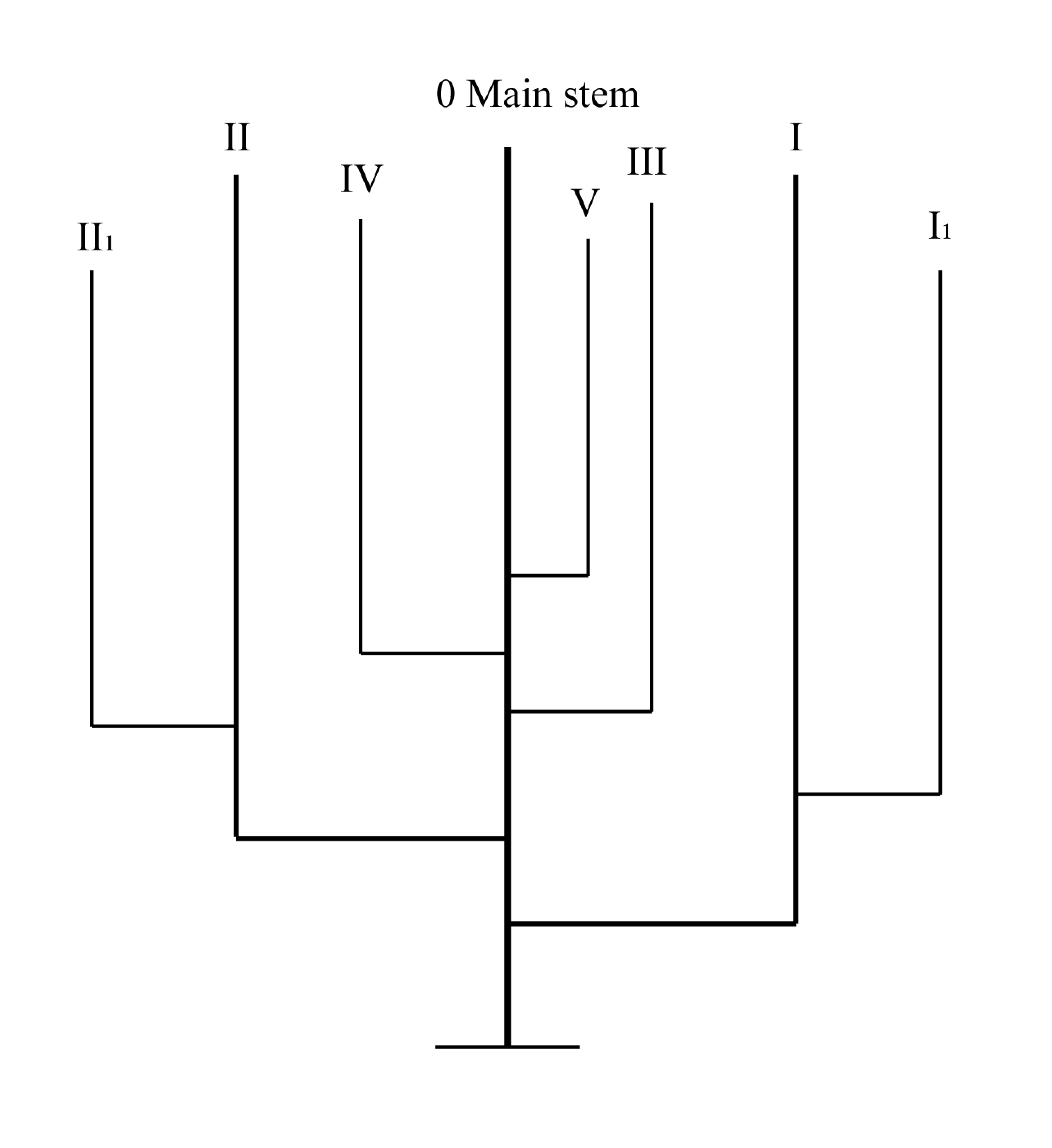

Supplement: Figure S3 — The main stem was denoted as 0; primary tillers on the main stem in emergence order were referred to as I, II, III, IV, and V. Secondary tillers on the primary tillers in emergence order were referred to as I 1 and II 1. [file peerj-07-6484-s003.png]

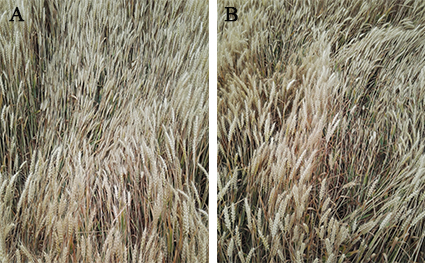

Supplement: Figure S4 [file peerj-07-6484-s004.jpg]
