# Supplementary material for: Optimizing plant density and nitrogen application to manipulate tiller growth and increase grain yield and nitrogen-use efficiency in winter wheat
Source: PeerJ. 2019 Feb 26;7:e6484. doi: 10.7717/peerj.6484 (PMC6396748; doi:10.7717/peerj.6484)
Supplement: Table S1 — GY, grain yield; SN, spike number; GN, grain number; GW grain weight. Correlation coefficients (r) are calculated and asterisks (∗∗) represent significance at the 0.01 probability level and asterisks (∗) represent significance at the 0.05 probability level. [file peerj-07-6484-s005.docx]

| Components | GY | SN | TGW | GN |
| --- | --- | --- | --- | --- |
| GY | 1 | 0.49 * | 0.27 | 0.03 |
| SN | 0.49 * | 1 | -0.62 ** | -0.74 ** |
| TGW | 0.27 | -0.62 ** | 1 | 0.92 ** |
| GN | 0.03 | -0.74 ** | 0.92 ** | 1 |

Table S1 Correlation analysis of grain yield and yield components.

GY, grain yield; SN, spike number; GN, grain number; GW grain weight. Correlation coefficients (*r*) are calculated and asterisks (**) represent significance at the 0.01 probability level and asterisks (*) represent significance at the 0.05 probability level.
