# Supplementary material for: Optimizing plant density and nitrogen application to manipulate tiller growth and increase grain yield and nitrogen-use efficiency in winter wheat
Source: PeerJ. 2019 Feb 26;7:e6484. doi: 10.7717/peerj.6484 (PMC6396748; doi:10.7717/peerj.6484)
Supplement: Table S2 — GY, grain yield; SN, spike number; GN, grain number; GW grain weight. Correlation coefficients (r) are calculated and asterisks (∗∗) represent significance at the 0.01 probability level. [file peerj-07-6484-s006.docx]

Table S2 Partial correlation analysis of grain yield and yield components.

| *r* (GY, SN) | | *r* (GY, TGW) | | *r* (GY, GN) | |
| --- | --- | --- | --- | --- | --- |
| Partial correlation coefficient | t-test | Partial correlation coefficient | t-test | Partial correlation coefficient | t-test |
| 0.81 ** | 5.11 | 0.70 ** | 3.68 | -0.24 | 0.93 |

GY, grain yield; SN, spike number; GN, grain number; GW grain weight. Correlation coefficients (*r*) are calculated and asterisks (**) represent significance at the 0.01 probability level.
