# Supplementary material for: Optimizing plant density and nitrogen application to manipulate tiller growth and increase grain yield and nitrogen-use efficiency in winter wheat
Source: PeerJ. 2019 Feb 26;7:e6484. doi: 10.7717/peerj.6484 (PMC6396748; doi:10.7717/peerj.6484)
Supplement: Table S3 — GY, grain yield; SN, spike number; GN, grain number; GW grain weight. [file peerj-07-6484-s007.docx]

Table S3 Path analysis of yield components and grain yield.

| Components | Direct effect | Indirect effect | | |
| --- | --- | --- | --- | --- |
|  |  | SN | TGW | GN |
| SN | 0.96 |  | -0.74 | 0.26 |
| TGW | 1.19 | -0.60 |  | -0.33 |
| GN | -0.36 | -0.71 | 1.09 |  |

GY, grain yield; SN, spike number; GN, grain number; GW grain weight.
